# Fast Projection Matching for X-ray Tomography

**DOI:** 10.1038/s41598-017-04020-0

**Published:** 2017-06-16

**Authors:** Chun-Chieh Wang, Cheng-Cheng Chiang, Biqing Liang, Gung-Chian Yin, Yi-Tse Weng, Liang-Chi Wang

**Affiliations:** 1 National Synchrotron Radiation Research Center, 30076 Hsinchu, Taiwan; 2 Department of Earth Sciences, National Cheng Kung University, 70101 Tainan, Taiwan; 3 Collection Management Department, National Taiwan Museum, 10047 Taipei, Taiwan

## Abstract

X-ray 3D tomographic techniques are powerful tools for investigating the morphology and internal structures of specimens. A common strategy for obtaining 3D tomography is to capture a series of 2D projections from different X-ray illumination angles of specimens mounted on a finely calibrated rotational stage. However, the reconstruction quality of 3D tomography relies on the precision and stability of the rotational stage, i.e. the accurate alignment of the 2D projections in the correct three-dimensional positions. This is a crucial problem for nano-tomographic techniques due to the non-negligible mechanical imperfection of the rotational stages at the nanometer level which significantly degrades the spatial resolution of reconstructed 3-D tomography. Even when using an X-ray micro-CT with a highly stabilized rotational stage, thermal effects caused by the CT system are not negligible and may cause sample drift. Here, we propose a markerless image auto-alignment algorithm based on an iterative method. This algorithm reduces the traditional projection matching method into two simplified matching problems and it is much faster and more reliable than traditional methods. This algorithm can greatly decrease hardware requirements for both nano-tomography and data processing and can be easily applied to other tomographic techniques, such as X-ray micro-CT and electron tomography.

## Introduction

X-ray nano-tomographic techniques are powerful tools for investigating the fine internal structures of specimens on a nanometer scale^1, 2^. However, precise projection image registration is crucial for the fidelity of specimen tomography reconstruction at nanometer spatial resolutions. When using X-ray micro-CT, near perfect sample positioning can be easily achieved using highly precise and stable rotational stages. However, with X-ray nano-CT or electron tomography, positioning precision is challenging due to the demanding structural details of specimens at nanometer level. Although projection misalignment caused by the mechanical imperfection of the rotational stage can be solved by precisely sensing and compensating for each rotational angle^3^, routine mechanical calibration is complex and time consuming. In addition, the sample may still drift due to thermal effects caused by stage motors^4^ or samples, which are heated by intense illumination during image acquisition.

To date, in X-ray nano-CT and electron tomography, the most reliable method is still placing nano-particles of high-contrast onto or near the targeted sample as reference points for image alignment. Using this method, projections can be aligned by either manual or automatic particle-tracking methods^5–9^. However, the manual alignment method is labor intensive and the extraneous high-contrast nano-particles are not always in the desired positions.

Over the past few decades, various markerless auto-alignment algorithms have been proposed to solve these problems, such as cross-correlation^10, 11^, common-line^12, 13^, feature matching^14–17^, and projection matching methods^18–20^. The precision of cross-correlation method relies on how short the rotational interval length is between two neighboring projections. However, the most common problem is that the cross-correlation methods do not consider real motion relations between objects through a full rotation. The other crucial problem is the subsequent accumulation of registration errors in correlated projections. The common-line method is well known for the corrections of in-plane rotation and vertical shift, yet inadequate in the correction of horizontal shift (supposedly the tilt axis is along the vertical axis). The common-line method uses the specimen center of mass to correct horizontal shift, which is not perfectly accurate and reliable unless the specimen is freely supported and the projections are well corrected in background. In contrast, the feature matching and projection matching methods do consider the real motion relations between objects by fitting geometric model and matching the re-projections of a guessed reconstruction model from various azimuth angles, respectively. However, large amount of statistics and calculation are needed which might demand high instrumental computation capacity and limit the throughput of the tomography reconstruction. Especially, in projection matching method, arguments remain regarding the fidelity of projection registration or the final reconstruction result.

One problem is that the fidelity of the tomography reconstruction relies on the quality of the initial projection alignment (or initial reconstruction). This means that a poor initial alignment may make it hard for the projection matching algorithm to converge, or it may only converge on a local extreme solution. In addition, correction of rotational axis tilt is another key issue for obtaining a good initial alignment. For example, Parkinson^18^ and Yang^21^ proposed a method to correct the rotational axis tilt by comparing the tilt difference between two projections obtained from 0° and 180° azimuth angles. This correction can roughly improve the accuracy of the initial alignment when using projection matching methods. Even so, the convergent process of traditional projection matching methods is still time consuming, and has demanding hardware requirements.

Here, we propose a new projection matching method that can gradually reduce the iterative computational complexity and increase the reliability of the optimized results by dividing a traditional projection matching problem into two simplified one-dimensional matching problems. This fast projection-matching algorithm (Faproma) is comprised of two correction processes. The first correction process simultaneously corrects vertical shifts and rotational axis tilts of raw projections using common-line concept, and the second process corrects horizontal shifts of raw projections using projection matching concept. This means that Faproma takes advantages of both common-line and projection matching methods. The improvements of Faproma are as follows: (1) prevent the unreliability of horizontal shift registration using traditional common-line methods; (2) reduce the complication of the projection matching process from 3D to 2D model projection.

## Methods

**Figure 1:**
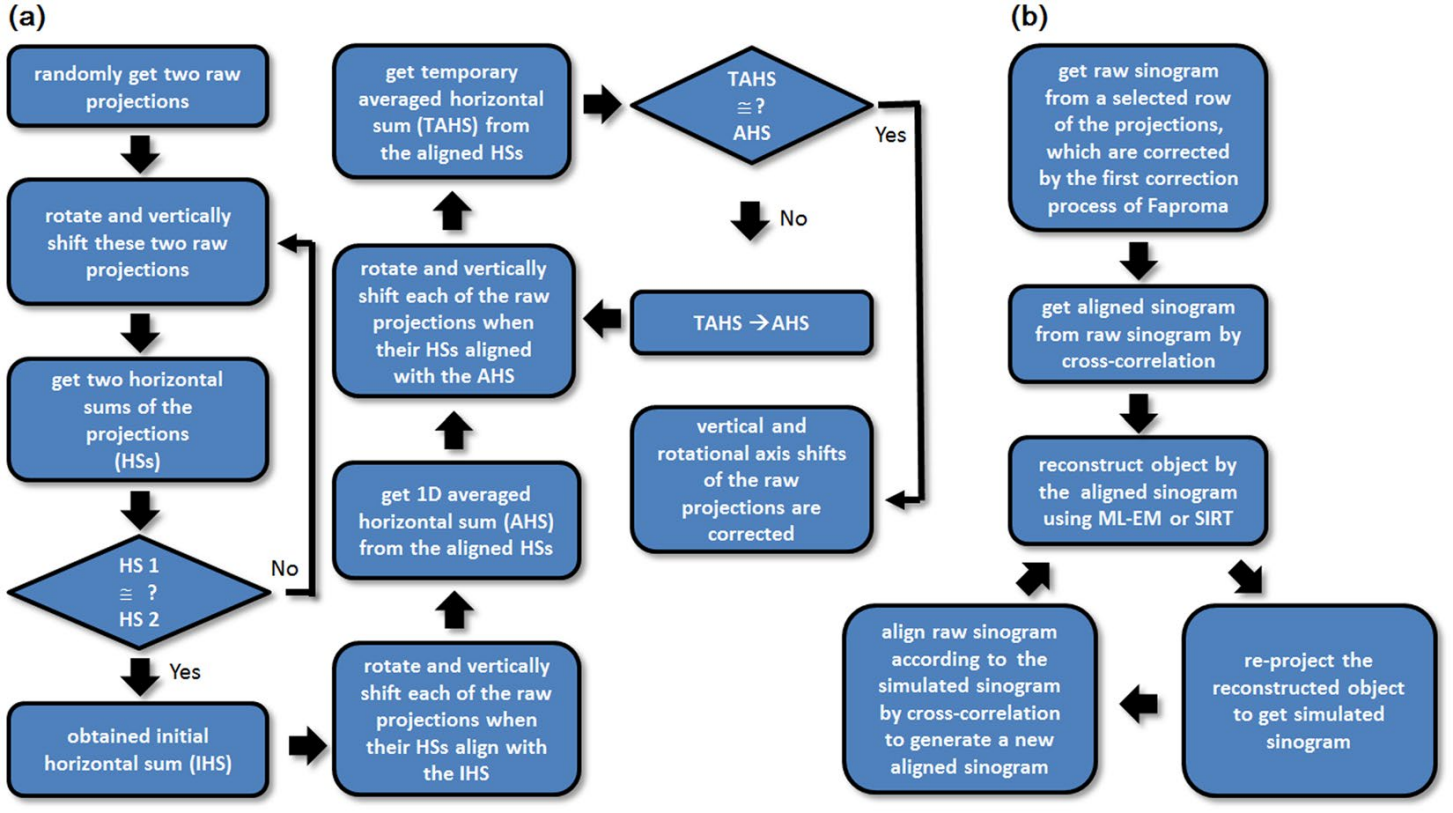
Flowchart of Faproma. (**a**) First correction process, which corrects vertical shift and rotational axis tilt simultaneously. (**b**) Second correction process, which corrects horizontal shift.

**Figure 2:**
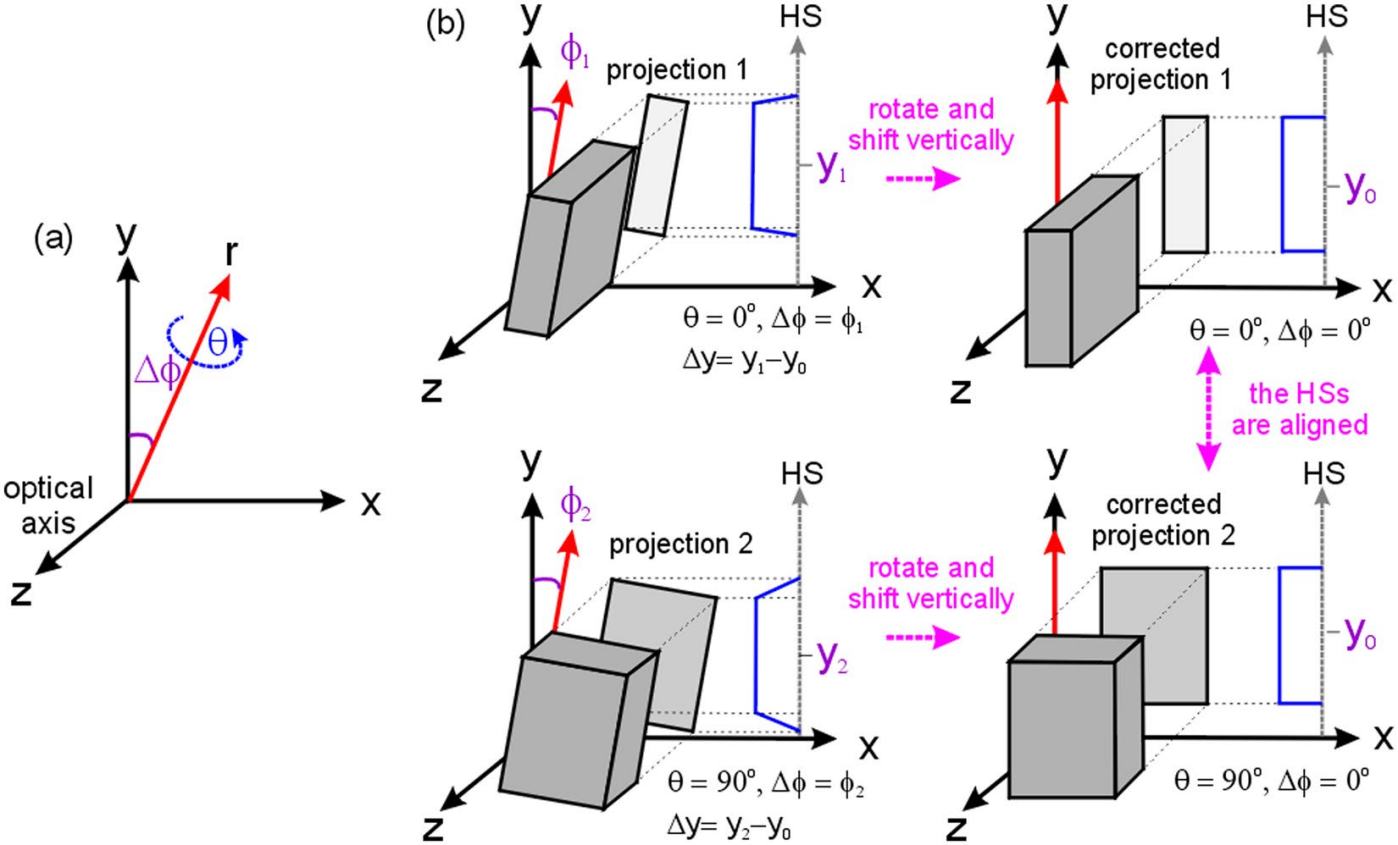
Method of vertical shift and rotational axis tilt corrections. (**a**) Spatial parameter definition. (**b**) Method for correcting two raw projections, which are obtained from different azimuthal angles (*θ* = 0° and 90°), with different rotational axis tilts (Δ*ϕ* = *ϕ*_1_ and *ϕ*_2_), and vertical shifts (Δ*y* = *y*_1−_ *y*_0_ and *y*_2−_ *y*_0_, where *y*_0_ is an arbitrary reference position). The blue line profiles in (**b**) show the corresponding horizontal sums (HSs). If the vertical shift and rotational axis tilt of raw projections, which are captured from different azimuth angles, are well corrected, each of their HSs will be the same as shown in (**b**).

The Faproma algorithm is demonstrated in the flowchart in Figure 1. The first correction process simultaneously corrects the vertical shift (Δ*y*) and rotational axis tilt (Δ*ϕ*) of raw projections (Fig. 1a). Under perfect conditions for finely-aligned projections, the sum of the intensity values on each row of projections captured from different azimuth angles, or defined here by the intensity sum as a horizontal sum (HS), will be the same in both position and intensity profile^22^. This concept originally comes from common-line method that proposed for electron-tomographic image registration^12, 13^. Using this hypothesis, we can randomly select two projections captured from different azimuth angles, tilt the rotational axes in series, and shift the y positions of these two projections until the HSs of these two projections align (see Fig. 2b). We can then obtain an initial horizontal sum (IHS) for the preliminary correction of vertical shift and rotational axis tilt for each projection obtained from different azimuth angles. However, real cases do not have such perfect conditions. A low signal to noise ratio and poor background correction of raw projections will both influence the HS profile and may lead to misalignments when using the preliminary IHS, which is calculated using only two projections. To overcome this complex problem, we use a similar vertical iterative alignment algorithm that proposed by Sanders and colleagues^22^ to obtain optimized vertical and rotational axis tilt corrections for each projection. The mathematical expression of the misaligned projections is as follows:

1 $${\tilde{p}}_{{\theta }_{i}}(f)(x,y,\varphi )\,{\rm{for}}\,i=1,2,\ldots ,k.$$

where *θ* is the azimuth angle, *f* is the 3D density function of the object, and *k* is the number of projections. The iterative algorithm for simultaneously correcting *y* and *ϕ* is as follows:

2 $$\begin{array}{c}argmin\\ {\rm{\Delta }}y,{\rm{\Delta }}\varphi \end{array}{\Vert {\int }_{R}{\tilde{p}}_{{\theta }_{i}}(f)(x,y-{\rm{\Delta }}y,\varphi -{\rm{\Delta }}\varphi )dx-\frac{1}{k}\sum _{l=1}^{k}{\int }_{R}{\tilde{p}}_{{\theta }_{l}}(f)(x,y,\varphi )dx\Vert }_{1}$$

where Δ*y* and Δ*ϕ* are corrections of vertical shifts and rotational axis tilts, respectively for the raw projections obtained from different azimuth angles (*θ*_*i*_). The averaged term, $\frac{1}{k}{\sum }_{l=1}^{k}{\int }_{R}{\tilde{p}}_{{\theta }_{l}}(f)(x,y,\varphi )dx$, here is called the averaged horizontal sum (AHS) and is used as the reference for the alignment of the raw HSs. A temporary AHS (TAHS), which is calculated from the average of newly aligned HSs, is used as a new AHS for the next iteration. The process has to be iterated multiple times until the TAHS is almost equal to the AHS.

**Figure 3:**
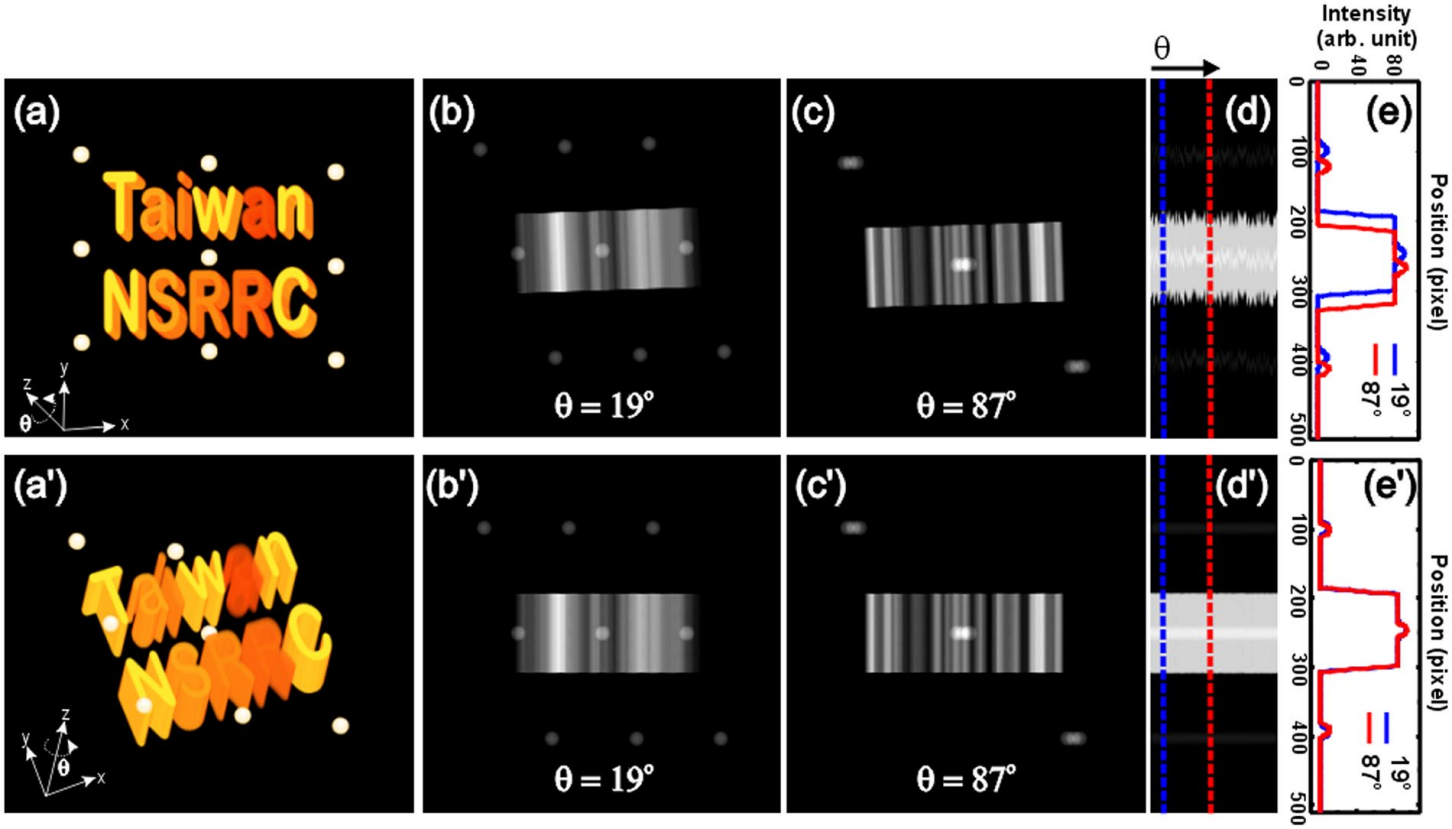
Demonstration of the Faproma’s first correction process using a computer-generated 3D phantom. (**a**) and (a’) show the artificial 3D object. (**b**) and (**c**) are simulated raw projections obtained from 19° and 87° azimuth angles, respectively, which were vertically shifted and rotated by computer in a random method. (**d**) The HSs obtained from 0° to 180° azimuth angles from the stimulated raw projections. The positions of the blue and red lines are the HSs, which were obtained from 19° and 87° azimuth angles, respectively. (**e**) The blue and red line profiles of the raw HSs are captured from projections, which were obtained from 19° and 87° azimuth angles, respectively. (b’) and (c’) are corrected projections obtained from 19° and 87° azimuth angles, respectively. (d’) Corrected HSs. The positions of the blue and red lines are the corrected HSs of projections, which were obtained from 19° and 87° azimuth angles, respectively. (e’) The blue and red line profiles are HSs, which were obtained from 19° and 87° azimuth angles, respectively, and were corrected by the Faproma’s first correction process. We can see these two HSs are aligned both in intensity profile and y position.

Figure 3 shows a test of the Faproma’s first correction algorithm by a computer-generated ideal 3D phantom (see Fig. 3a and f). We simulated a series of projections from different azimuth angles of the phantom that is similar to a series of projections we can capture using a transmission X-ray microscope. We then randomly shifted the projections in vertical and horizontal directions and slightly tilted them at random angles relative to the right rotational axis to simulate the rotational instability caused by the rotational stage or thermal effects. Figure 3b and c show the raw, randomly-shifted, and rotated projections captured at two randomly selected azimuth angles: 19° and 87°, respectively. Figure 3g and h show the corrected results of the two raw projections after using the first process of Faproma. Figure 3d and i compare the raw and finely-aligned HSs patterns of the test phantom. Figure 3d lists the HSs of 181 raw projections, which were obtained from 0° to 180°. Line profiles of the HSs captured at the randomly selected 19° (blue dashed line) and 87° (red dashed line) are shown in Figure 3e. We can clearly see mismatches in both intensity profiles and *y* positions of these two HSs. Figure 3g and h show the corrected projections obtained at 19° and 87° azimuth angles by the vertical and tilt correction process mentioned above. Figure 3i and j show the corrected HSs from 0° to 180° and HS line profiles obtained at 19° and 87° azimuth angles, respectively. We can see that both vertical shifts and rotational axis tilts of the raw projections are simultaneously corrected using this first correction process. Of note, only one iteration process is needed when the projections are obtained from an ideal phantom, which refers to conditions where there is no noise or background correction problems.

**Figure 4:**
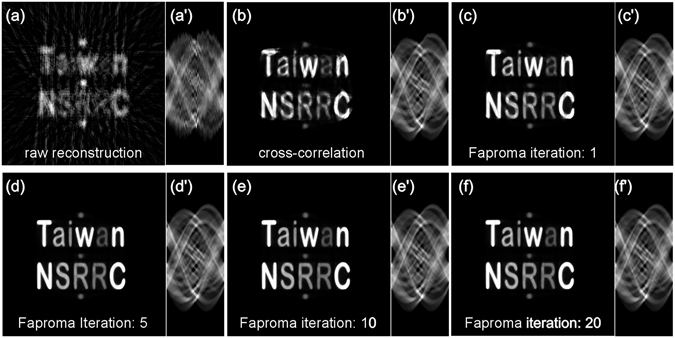
Demonstration of the second correction process using a computer-generated 3D phantom as shown in Fig. 3a and a’. (**a**) and (a’) are the selected reconstruction layer and its corresponding sinogram of the raw artificial projections, which were pre-corrected using the Faproma’s first correction process, respectively. (**b**) and (b’) are the layer reconstruction and corresponding sinogram after cross-correlation correction. (**c**–**f**) Are layer reconstruction results after 1, 5, 10, and 20 iterations of correction using the Faproma’s second correction process. (c’–f’) are the corresponding sinograms of (**c–f**).

After the Faproma’s first correction process, the only remaining problem of the registration is the correction of the horizontal shift of each projection. The horizontal registration can be considered on only one randomly selected layer (parallel to the *x*-*z* plane) when the vertical shifts and rotational axis tilts had been corrected. In brief, the problem is how to align each row of a raw sinogram, which is obtained from selected layer, to its correct horizontal position. As shown in Fig. 1b, the first step of the second correction process is to get pre-alignment of the raw sinogram corresponding to the selected layer using the one-dimensional cross-correlation method (see Fig. 4b). In this way, the pre-aligned sinogram will become an aligned sinogram. The second step is to use the aligned sinogram to get a two-dimensional reconstruction with an iterative reconstruction algorithm, such as the maximum-likelihood estimation method (ML-EM)^23^,or simultaneous iterative reconstruction technique (SIRT)^24^. The reconstruction obtained by these methods will be the optimal solution for the aligned sinogram. The third step is to re-project the two-dimensional reconstruction to get a simulated sinogram. The fourth step is to align each raw sinogram row according to the simulated sinogram to get a new aligned sinogram. The final step is to repeat the second step until the aligned sinogram closely matches the simulated sinogram. This sinogram alignment using projection-matching iterations allows us to create an optimized reconstruction for the selected layer of the object and correct for horizontal shifts in the raw projections.

We can get accurate translational shifts and rotational axis tilts of raw projections using these two simple correction processes and gradually reduce computer processing time compared to the traditional projection matching methods used in soft X-ray tomography^25^ that simultaneously deal with three degrees of freedom in the *x*, *y*, and *ϕ* dimensions. The Faproma can process projection registration more efficiently than the traditional projection matching method for two reasons. The first is that the translational correction between raw projection and corresponding re-projection is estimated by using a two-dimensional cross-correlation method in a traditional projection matching method. However, the two-dimensional cross-correlation method works very well when two images of an object only differ in lateral shift: it does not work well when the raw projection of the object and the corresponding re-projection of the guess reconstruction are significantly different in morphology as the guess reconstruction is far from the real solution. Morphological differences between the raw projection and the corresponding re-projection may lead to a correlation error in both the x and y dimensions using the two-dimensional cross-correlation method. In brief, the corrections both in x and y directions will be implicative of each other. This may impair the traditional projection matching algorithm from converging or render possibly converging on a locally extreme solution. That is why the alignment reliability of the traditional projection-matching method is related to the quality of the initial reconstruction or initial alignment. In contrast, Faproma corrects translational shift in the x and y dimensions using different mechanisms and the Faproma iterative algorithm can therefore converge much more quickly and easily. This means that algorithm iteration times can be substantially reduced. The second reason is that in each iteration process the traditional projection matching method has to temporarily reconstruct whole layers of a 3D object for next re-projection, but Faproma only has to reconstruct one selected layer, which dramatically reduces total processing time for projection registration. A factor of the processing time (*t*_factor_ = *t*_pm_/*t*_Faproma_) between the traditional projection matching method (*t*_pm_) and Faproma (*t*_Faproma_) is proportional to the layer number of the 3D reconstruction (*N*_layer_) and the difference in iteration times between the traditional projection matching method (*N*_iter-pm_) and Faproma (*N*_iter-Faproma_). This can be simply written as: *t*_factor_ α *N*_layer_ × (*N*_iter-pm_ − *N*_iter-Faproma_). It is clear that the total processing time of Faproma is dramatically shorter than the traditional projection matching method, especially in cases of large image size.

Figure 4 shows the performance of the Faproma’s second correction process. Figure 4a and a’ show the reconstruction and its sinogram corresponding to a selected layer which was obtained from simulated raw projections. The vertical shifts and rotational tilts of the simulated raw projections were corrected using the Faproma’s first correction process. Without applying any horizontal shift correction, the raw reconstruction is very blurry. The horizontal misalignment can be clearly seen from the raw sinogram (Fig. 4a’). We also compared the registration quality of the raw sinogram between cross-correlation method (Fig. 4b) and the Faproma’s second correction process (Fig. 4c–f). After sinogram registration using the cross-correlation method, the new reconstruction is better than the raw reconstruction (Fig. 4a), but it is still not optimal. We can see that the reconstruction quality is much better than the cross-correlation result after just one iteration process using the Faproma’s second correction process (see Fig. 4b,c). Typically, reconstruction quality can almost reach optimization within only ten iterations using the Faproma’s second correction process. (see Fig. 4c–f).

## Results

**Figure 5:**
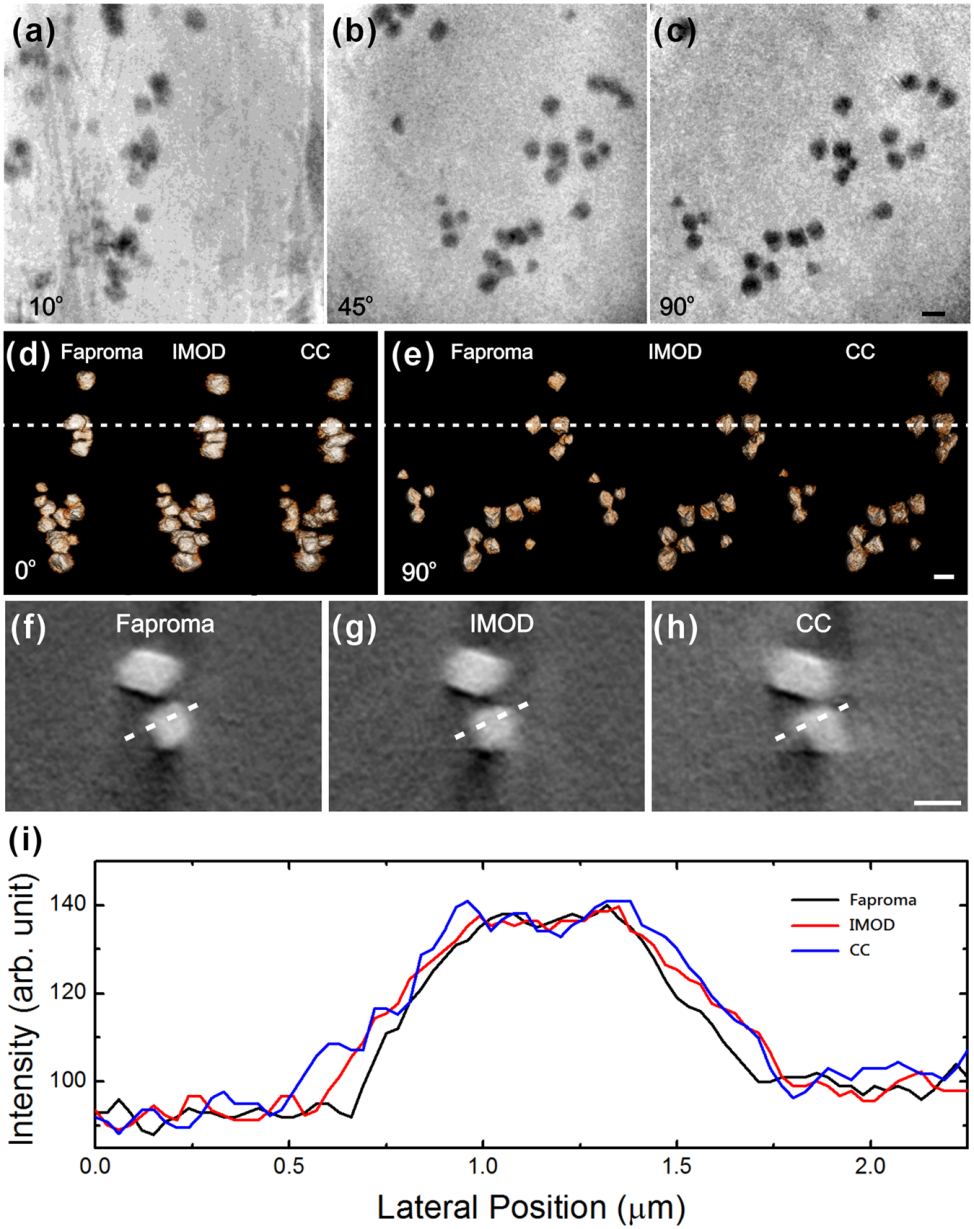
Synchrotron transmission X-ray microscopy (TXM) data processed by Faproma, IMOD and cross-correlation (CC) methods for correction comparison. (**a–c**) Raw TXM data obtained at 10°, 45°, and 90° azimuth angles. (**d–e**) 3D tomographies of globular pyrites observed from 0° and 90° azimuth angles that were reconstructed from projections, and corrected using the Faproma, IMOD, and CC methods, respectively. (**f–h**) Sections of the globular pyrite 3D tomographies, which were reconstructed using Faproma, IMOD, and CC correction, respectively. The 3D tomography by the Faproma method shows clearer boundaries than those by the IMOD and CC methods. In the IMOD case, 22 pyrite particles were tracked for alignment calculation. Comparisons of alignment and reconstruction quality among three methods are shown in Supplementary Movies S1 and S2. Line profiles were obtained along the white-dashed lines in (**f–h**). (**i**) Comparison of tomography resolution influences between the Faproma, IMOD, and CC methods. The line profile of the pyrite was obviously narrower and the edge response was sharper in the Faproma than that in IMOD and CC methods, which pointed to better projection registration and subsequent superior reconstruction quality and tomography resolution. The 3D rendering is shown using ORS Visual SI software (Object Research Systems Inc., Montreal, Quebec, Canada). Scale bar: 1 μm.

We demonstrated the performance of Faproma for real projections, which were obtained by a synchrotron transmission X-ray microscope (TXM) at BL01B1 beamline of Taiwan Light Source (TLS). The X-ray projections of globular pyrite structures from black shale^26^ were reconstructed after registration using Faproma, IMOD, and cross-correlation methods (see Fig. 5d,e), respectively. IMOD is a popular open-source software for CT image registration and reconstruction based on multi-particle tracking method^6^. In comparison, the resulted images from Faproma illustrated clearer and sharper boundaries for the minerals than those from the other two methods (see Fig. 5f–h and Supplementary Movies S1 and S2). Better outlined boundaries attribute to a higher reconstruction quality and fidelity by Faproma. In case of the shape and size of tracking markers, particle tracking works well mostly for markers with near spherical shape or small enough, and the resulted projection morphology of the markers are nearly un-changed during the rotation. However, particle-tracking based image registration is not suitable for the alignment of pyrite dataset when the octahedron-shaped pyrites are used as an intrinsic fiducial marker. The reason is that the center of mass of a non-rotational symmetric marker in the projection plane will be hard to define when the marker is overlapped with other features in the projection plane. Based on the comparison of tomography resolution influences, the line profile of the pyrite was obviously narrower and the edge response was sharper in the Faproma than that in IMOD and cross-correlation methods (Fig. 5i), which pointed to better projection registration and subsequent superior reconstruction quality and tomography resolution. It is evident that the registration method performance is closely related to the quality and spatial resolution of the reconstructed tomography.

**Figure 6:**
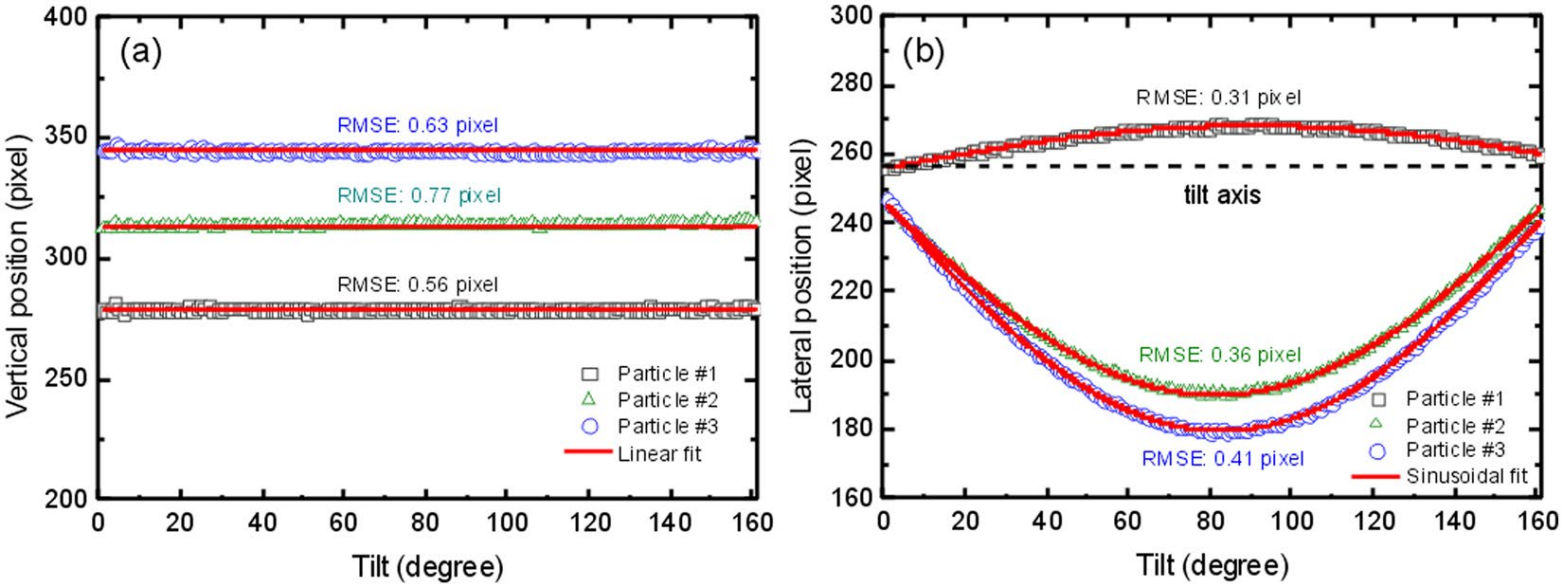
Evaluation of Faproma registration accuracy. (**a**) Linear fits with vertical positions of three gold nanoparticles obtained in projections aligned using Faproma. (**b**) Sinusoidal fits with horizontal positions of three nanoparticles obtained in projections aligned using Faproma. The nanoparticle positional tracking on each projection was performed using the MTrack2 plugin module in ImageJ software. The alignment result is shown in Supplementary Movie S3. The image size of each projection is 512 × 512 pixels, and each pixel is corresponding to 30 nm on the object plane. RMSE: root mean square error.

The alignment accuracy of Faproma was also evaluated using gold nanoparticle projection dataset obtained using the TXM. We use small and nearly spherical gold nanoparticles as the specimens, as they are easier to track the positions of particles in each projection according to their center of mass on the image plane. We ensure that the nanoparticles are freely supported and the projections are well background corrected, thus the position identifications of the nanoparticles are theoretically accurate. By fitting linear and sinusoidal functions on the vertical and horizontal positions of nanoparticles in Faproma aligned projections, we get the deviation of the aligned nanoparticle motions relative to the theoretical point motions that validates the accuracy of alignment^9^. We selected three free-supported nanoparticles for evaluation. The vertical root-mean-square-errors of these three nanoparticles between the aligned positions and linear fitting curves are about 0.56, 0.77, and 0.63 pixel (corresponding to 19.5, 23.1, and 18.9 nm in real space), and the horizontal root-mean-square-errors of these three nanoparticles between the aligned positions and sinusoidal fitting curves are about 0.31, 0.36, and 0.41 pixel (corresponding to 9.3, 10.8, and 12.3 nm in real space), as shown in Fig. 6 and Supplementary Movie S3. Such extremely small root-mean-square-errors reflect negligible shift from the theoretical point during alignment, fall far below the spatial resolution limit of the most advanced TXM instrument, and thus provide evidence that our Faproma method is highly reliable.

**Figure 7:**
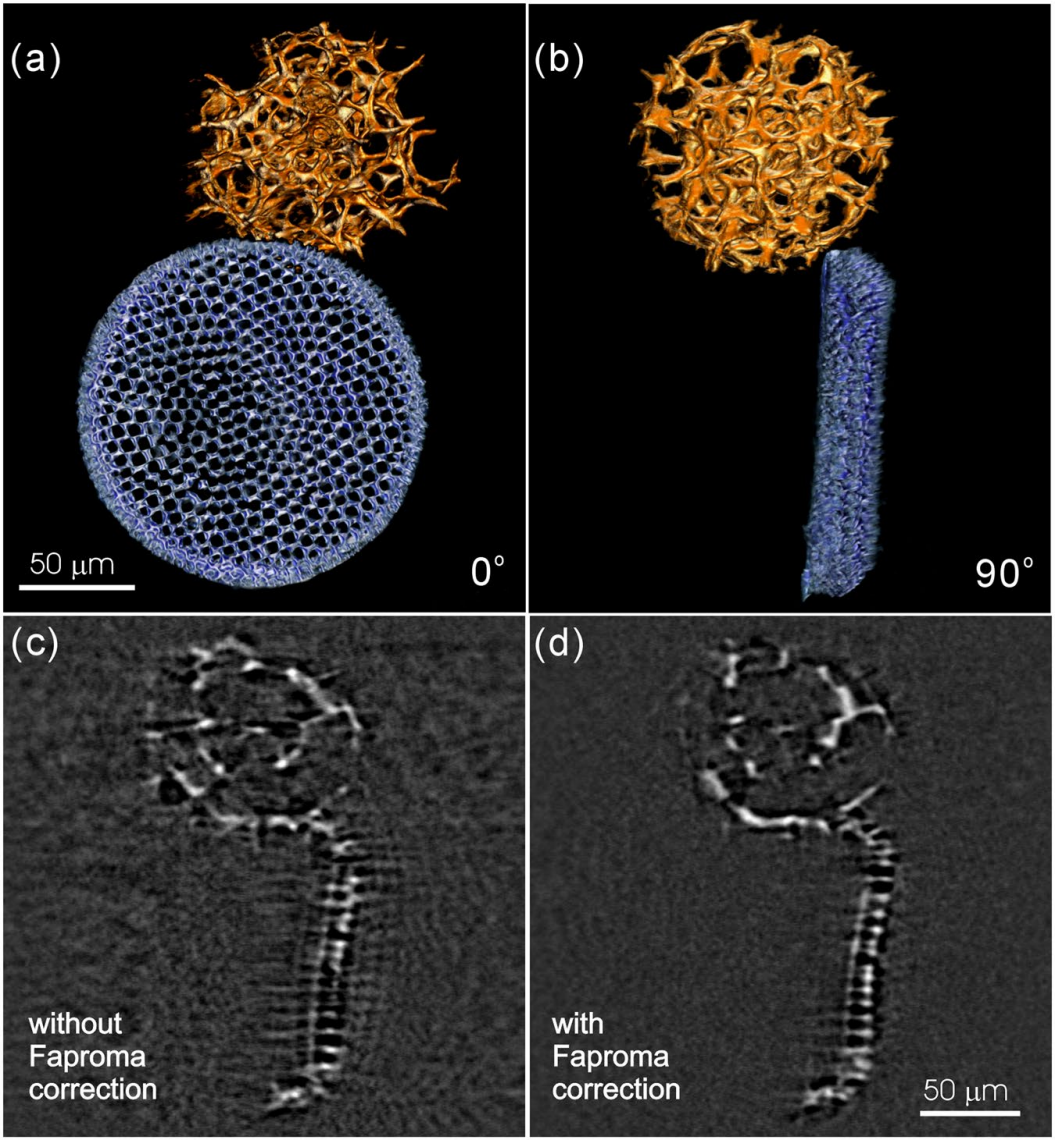
Synchrotron X-ray micro-CT data corrected using Faproma. (**a,b**) Well corrected 3D tomography of micro-fossils at 0° and 90°. (**c**) Reconstructed section without applying any registration method. (**d**) Reconstructed section after Faproma registration. We can see that the boundary of the reconstructed section became much clearer and most of the artifacts disappeared after the Faproma correction. The reconstruction quality comparison is also shown in Supplementary Movie S4. The 3D rendering is shown using ORS Visual SI software (Object Research Systems Inc., Montreal, Quebec, Canada).

In addition, Faproma is also valuable for X-ray micro-CT applications owing to the issue of thermal effects in tomographic reconstruction, particularly for high flux synchrotron-based X-ray micro-CT. Specimens or specimen supporting materials, such as epoxy and Kapton polyimide film, which are commonly used in TXM and X-ray micro-CT applications, expand after being heated by high-flux X-ray illumination and this expansion may shift specimen position during imaging. To solve this problem, it is necessary to leave a few seconds waiting time between two sequential acquisitions to decrease the accumulated heat effects within specimens and specimen supporting materials. However, total tomography acquisition time will be dramatically increased by this waiting requirement, which is a matter for time-resolved CT systems^27^. To enhance tomography quality and increase the data acquisition throughput of X-ray micro-CT, we propose correcting positional shifts of specimens due to thermal effects using Faproma. To test X-ray micro-CT applicability, we attached microfossils to Kapton polyimide film and acquired their tomography using a synchrotron-based X-ray micro-CT at the BL01A1 beamline of TLS without incurring any waiting time between acquisitions. Figure 7 shows the 3D tomography of microfossils obtained by X-ray micro-CT. The dish-like microfossil is a valve of the marine diatom *Coscinodiscus oculusiridis* (Ehrenberg) Ehrenberg, while the spherical structure is a fragment of radiolarian exoskeleton. Such microfossils which have sub-micrometer porous structures are often used to check the spatial resolution of optical microscopes. Figure 7c shows a section of the internal structure of the microfossil that was directly reconstructed according to the raw projection data set; Fig. 7d shows the corresponding section of the internal structure of the microfossil that was reconstructed after registration by Faproma. It is apparent that thermal effects decreased the spatial resolution and fidelity of the X-ray micro-CT tomographic reconstruction and increased reconstruction artefacts (Fig. 7c). By contrast, Faproma was an effective approach for calibrating and remediating the positional errors caused by thermal effect (see Fig. 7d and Supplementary Movie S4).

## Discussion

Markerless image registration is crucial for computed tomography, especially when the spatial resolution of the image system exceeds the stability of the mechanical motion system, especially with regards to X-ray nano-CT and electron tomography systems. The projection matching method is a useful technique for addressing this problem. There are some disadvantages which limit the application of traditional projection matching methods, such as inefficiency, demanding hardware requirements, and the dubious fidelity of reconstructions. For example, the traditional projection matching method applied in a synchrotron soft X-ray tomography system^18^ needed an approximately 35s calculation time for one matching iteration with 128 parallel processors. The iteration requirement for an acceptable 3D reconstruction using this method are typically more than 50 times, which means that the total processing time for one fine 3D tomography result will be longer than thirty minutes on a large computer cluster system. As shown in the literature, one would need more than 22 hours on a personal computer with one processor to calculate one tomography dataset composed of 91 projections with a 500 × 800 pixel resolution.

**Figure 8:**
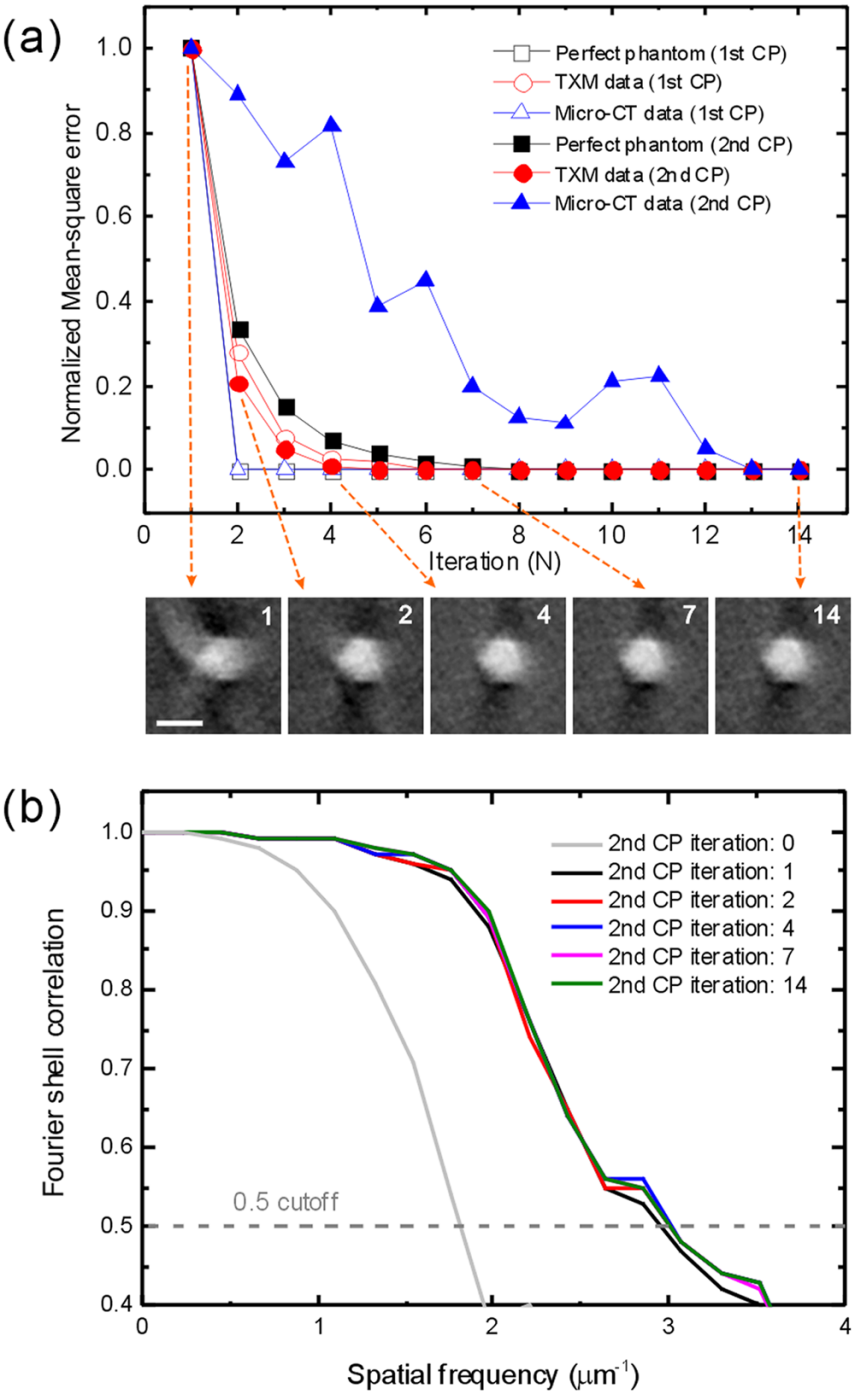
Convergence of Faproma algorithm. (**a**) Comparison of the normalized mean-square error and iteration times among various 3D tomography datasets. The inserted images show the resulted reconstruction quality with different registration iteration times using Faproma second correction process (see Supplementary Movie S5). (**b**) Three-dimensional Fourier shell correlation among different iteration times by the second correction process of Faproma. CP: correction process. Scale bar is 1 μm.

Table 1(b) shows the computational time and iteration times to process one real tomographic dataset. The dataset was obtained using the TXM and was composed of 161 projection images with a 512 × 512 pixel resolution using Faproma. The reconstruction quality defined by normalized mean-square errors between the aligned sinogram and the simulated sinogram reached its optimization within five and seven iterations using the first and second correction processes, respectively (see Fig. 8a). The optimization criterion is defined as a normalized mean-square error bellow 1/e. The total computational time was less than 20 minutes using only a single computer (Z600, HP) with two 2.4 GHz processors. Therefore, this algorithm can be easily applied on most desktop computers and has a reasonable processing time. We also compare the reconstruction quality of different iteration times after Faproma second correction process. The reconstruction quality was evaluated using three-dimensional Fourier shell correlation (FSC) method^28, 29^ (see Fig. 8b). According to a 0.5 cutoff, for different FSC curves with various iterations, optimization was reached after only 2 iterations, and the curve extended to high spatial frequency.

**Table 1:** Comparison of iteration times, calculation times, and hardware requirements for data obtained using various techniques and registration methods.

Case	(a) Perfect phantom	(b) TXM data	(c) Micro-CT data	(d) Ref. 16
Registration method	Faproma	Faproma	Faproma	PM
Data size	512 × 512 pixels (181 images)	512 × 512 pixels (161 images)	701 × 701 pixels (89 images)	800 × 500 pixels (90 images)
Calculation time for the first correction process	269.3 s (1 iteration)	565.9 s (5 iterations)	53.5 s (1 iterations)	—
Calculation time for the second correction process	682.2 s (7 iterations)	526.8 s (7 iterations)	765.5 s (12 iterations)	>40000 s (>50 iterations)
Number of CPU processors	2	2	2	2
Total calculation time	951.5 s	1092.7 s	818.8 s	>40000 s

The total calculation time in (b) is longer than in (a) because the signal-to-noise ratio of real cases will influence the total calculation time of Faproma. The calculation time for the first correction process is shorter in (c) than in (b) due to the quality of the background correction. The signal-to-noise ratio of the micro-CT data is better than for the TXM data. PM: traditional projection matching.

The Faproma algorithm is simply coded based on Matlab (Matlab R2014b, The MathWorks, Inc.) in this work for a quick demonstration. Yet, the algorithm speed may still be increased by algorithm optimization, or through using a graphics processing unit (GPU) to run the calculation. The most important advantage of the Faproma registration algorithm is that it dramatically reduces the hardware requirements for both data processing and tomography acquisition, alleviating the need for ultrahigh stable motion components in tomographic systems.

## Electronic supplementary material

Supplementary Information

Supplementary Movie S1

Supplementary Movie S2

Supplementary Movie S3

Supplementary Movie S4

Supplementary Movie S5
