# Supplementary material for: Fast Projection Matching for X-ray Tomography
Source: Sci Rep. 2017 Jun 16;7:3691. doi: 10.1038/s41598-017-04020-0 (PMC5473817; doi:10.1038/s41598-017-04020-0)
Supplement: Supplementary file 1 — Supplementary Information [file 41598_2017_4020_MOESM1_ESM.pdf]

Supplementary Information

Fast Projection Matching for X-ray Tomography

Chun-Chieh Wang[1]*, Cheng-Cheng Chiang[1], Biqing Liang[2]**, Gung-Chian Yin[1], Yi-Tse Weng[2], and Liang-Chi Wang[3]

[1]National Synchrotron Radiation Research Center, 30076 Hsinchu, Taiwan.

[2] Department of Earth Sciences, National Cheng Kung University, 70101 Tainan, Taiwan.

[3] Collection Management Department, National Taiwan Museum, 10047 Taipei, Taiwan.

CORRESPONDING AUTHOR

Chun-Chieh Wang (E-mail: wang.jay@nsrrc.org.tw)

CO-CORRESPONDING AUTHOR

Biqing Liang (E-mail: betacau07@gmail.com)

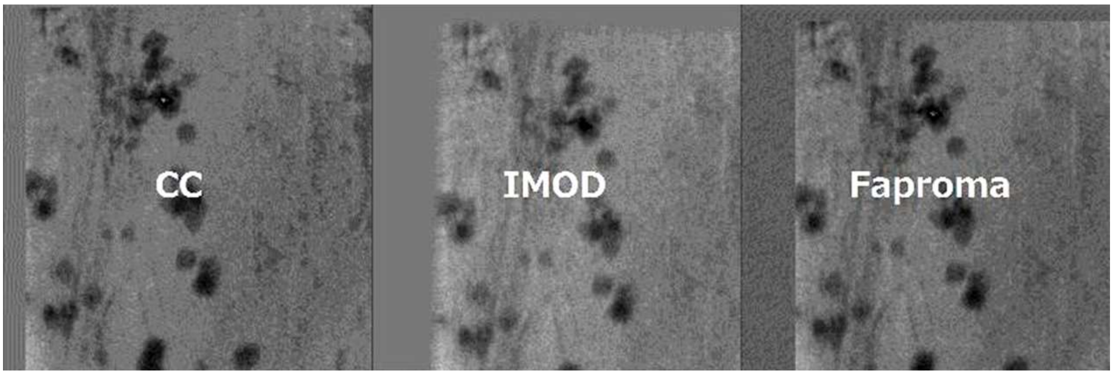

Supplementary Movie S1. Comparison of alignment quality among cross-correlation, IMOD, and Faproma methods.

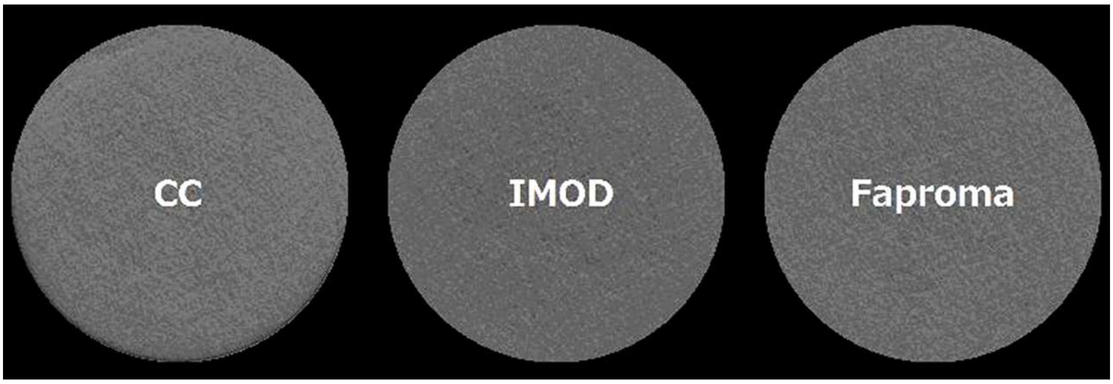

Supplementary Movie S2. Comparison of reconstruction quality among cross-correlation, IMOD, and Faproma methods.

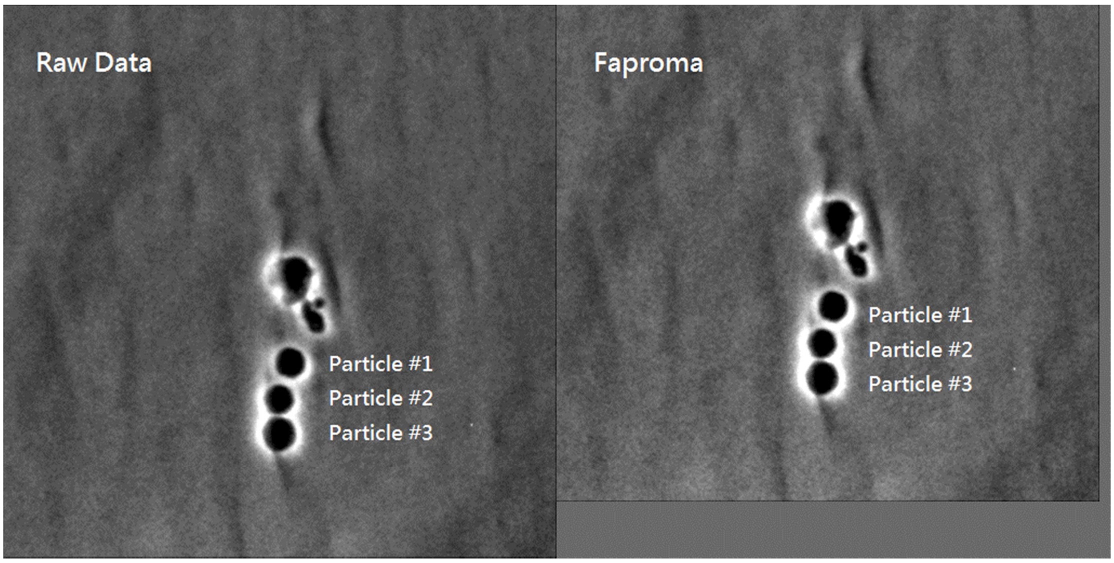

Supplementary Movie S3. Alignment accuracy evaluation of Faproma.

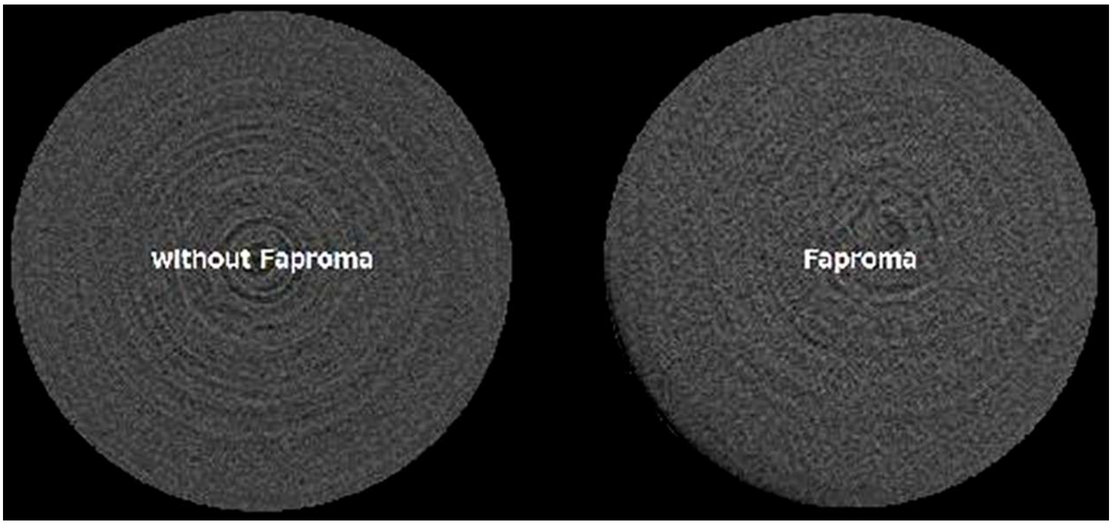

Supplementary Movie S4. Comparison of reconstruction quality between with and without image registration using Faproma.

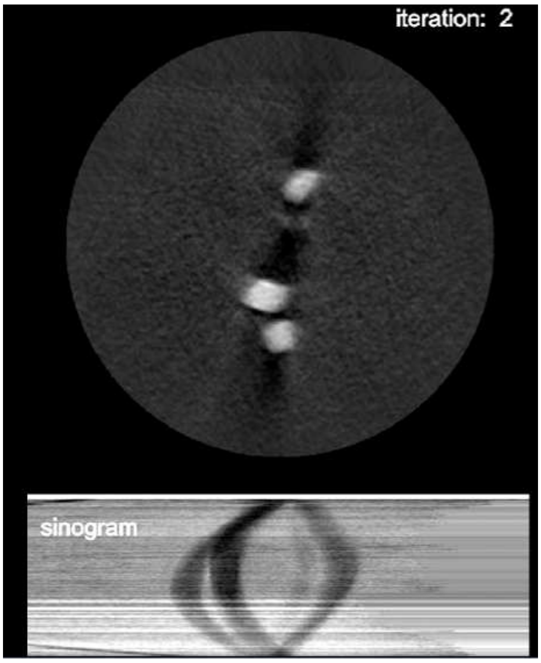

Supplementary Movie S5. Comparisons of reconstruction and sinogram alignment quality during the iteration process of Faproma.
